# Acute Perforated Appendicitis Complicated by Necrotizing Fasciitis and Bladder Perforation

**DOI:** 10.7759/cureus.12764

**Published:** 2021-01-18

**Authors:** John Oh

**Affiliations:** 1 Emergency Department, Kent Hospital, Warwick, USA

**Keywords:** case report, appendicitis, necrotizing fasciitis, bladder perforation

## Abstract

Acute uncomplicated appendicitis is a common surgical disease that has been well-studied, and its overall mortality has decreased over time. However, delay in treatment can be associated with rare complications such as necrotizing fasciitis, which carries a high mortality rate, and bladder perforation. We present such a case in an 81-year-old female with no significant surgical history who presented to the emergency department with four days of abdominal pain. A CT scan revealed extensive subcutaneous air in the abdominal wall, an inflamed appendix, and a periappendiceal abscess. During subsequent exploratory laparotomy, she was also found to have bladder perforation. She underwent debridement of necrotic tissue of the abdominal wall, appendectomy, drainage of periappendiceal abscess, and bladder perforation repair. She died of septic shock on post-operative day 19, due to gross spillage of urine into the abdomen and ongoing necrotizing fasciitis. Acute perforated appendicitis can lead to rare and fatal complications. Our case presents such a patient with a poor outcome. In approaching a patient with signs of peritonitis, differential diagnoses must remain broad to include late complications such as abscess formation, soft tissue infection, and perforation of surrounding structures.

## Introduction

Acute appendicitis is a common surgical disease that is well-studied with an overall mortality rate of 1% [1]. However, it can lead to perforation and subsequent complications such as periappendiceal abscess or peritonitis, especially if disease recognition and management are delayed. In the case of perforation, the mortality increases nearly six-fold [2]. Complications that follow perforation are rare. Such cases are therefore frequently treated on a case-by-case basis without definitive evidence behind each step in management [3]. Necrotizing fasciitis is such a complication of acute appendicitis. It has an estimated mortality rate that widely varies between 6%-76% [4]. According to our search of the literature, there have been 15 reported cases associated with acute appendicitis, with a mortality rate of 33.3% (5/15) [5]. An even rarer complication is perforation of the bladder wall, of which we found three similar cases in the literature [6]. We report a case of perforated appendicitis causing necrotizing fasciitis of the abdominal wall and perforation of the bladder. Although our patient eventually died from her disease process, this case highlights how acute appendicitis can lead to rare complications where there are no established standards of management.

## Case presentation

An 81-year-old female with a past medical history significant for coronary artery disease, active left femoral vein deep vein thrombosis on warfarin, hypothyroidism, atrial fibrillation, and type 2 diabetes presented to the emergency department from a skilled nursing facility for evaluation of abdominal pain. The patient had been experiencing four days of abdominal pain, nausea, vomiting, and diarrhea. She noted her vomitus had a feculent odor. No blood was noted in the patient's emesis or bowel movements. The patient reported that "food goes right through me".

On physical examination, the patient was ill-appearing with a heart rate of 150/min in rapid atrial fibrillation, respiratory rate of 22/min, blood pressure of 100/60 mmHg, and oral temperature of 37.5°C. Abdominal exam was consistent with peritonitis with guarding in all four quadrants of the abdomen, which was most significant in the right lower quadrant. Of note, there was no palpable crepitus or overlying skin changes. Resuscitation was initiated with two liters of normal saline IV fluids.

**Figure 1:**
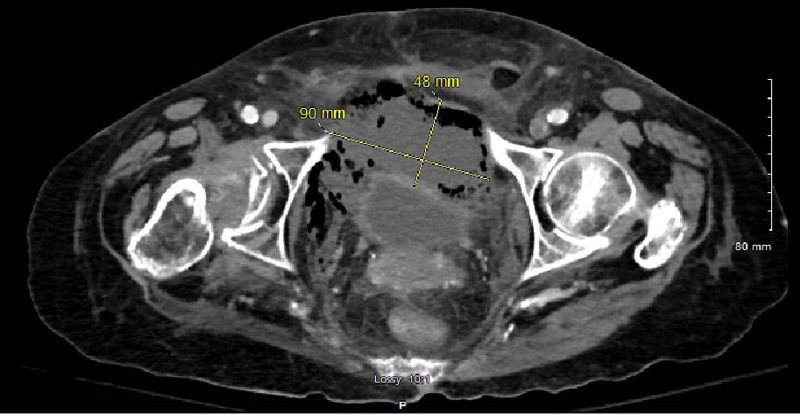
CT scan – axial view – the periappendiceal abscess was approximately 9.0 x 4.8 cm in size

**Figure 2:**
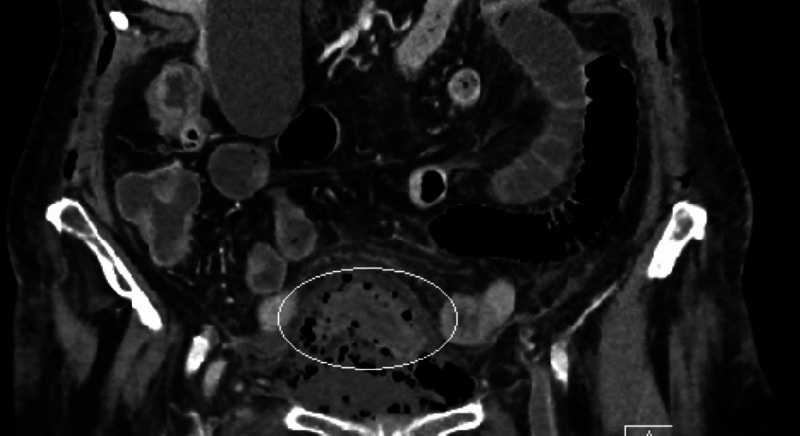
CT scan – coronal view – the gas from the periappendiceal abscess encircles the entire urinary bladder

**Figure 3:**
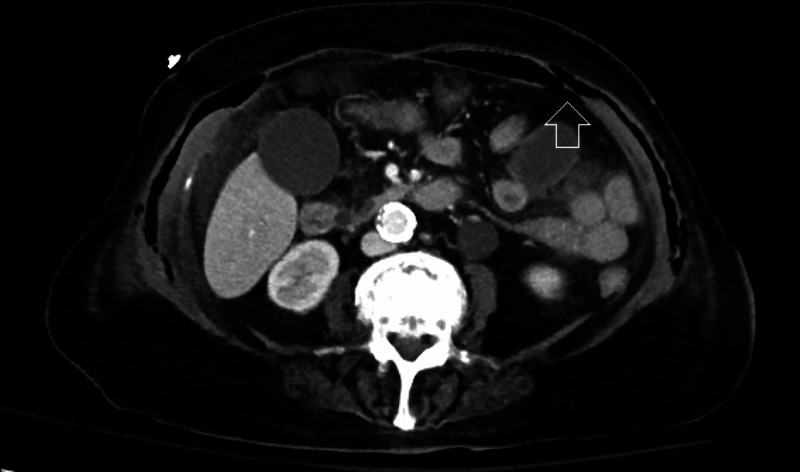
CT scan – axial view – demonstration of subcutaneous air; extensive gas is seen within multiple layers of the abdominal fascia, with the deepest affected layer immediate superficial to the parietal peritoneum (white arrow)

**Figure 4:**
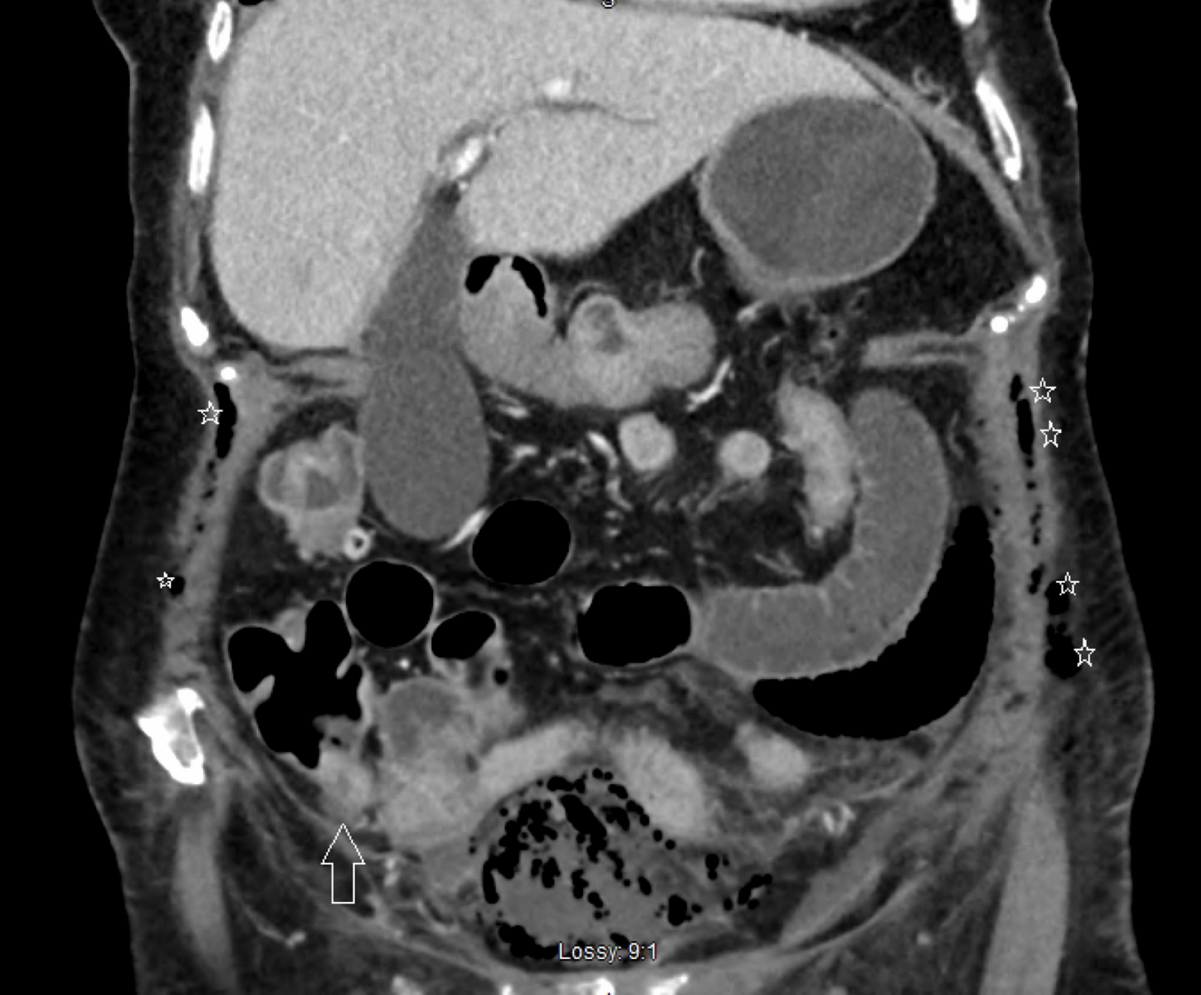
CT scan – coronal view – a thickened appendix (white arrow) with subcutaneous emphysema seen on both sides of the abdominal wall (white stars)

Labs demonstrated leukocytosis to 19,600/μL, hemoglobin of 9.0 g/dL, and a platelet count of 370,000/μL. Her international normalized ratio (INR) was 2.6. A metabolic panel was significant for creatinine 1.56, a significant increase from baseline. Her lactic acid was elevated at 4.0. An abdominal CT scan with intravenous contrast was obtained that showed a 9.0 x 4.8 cm fluid collection and gas anterior to the bladder (Figures 1-2). The gas appeared to track upward into the anterior abdominal wall with extensive subcutaneous gas present (Figure 3). Also noted were thickening of the cecum and appendix and bilateral hydronephrosis and hydroureter (Figure 4).

General surgery service was consulted for emergent laparotomy after anticoagulation reversal. She was given four units of fresh frozen plasma and vitamin K 10 mg IV for warfarin reversal, and ceftriaxone 1 g IV, and metronidazole 500 mg IV.

Exploratory laparotomy revealed unusual findings. Upon entrance into the abdominal wall, there was clear separation of the peritoneum from the overlying necrotic transversalis fascia extending down into the pelvis. A dense fibrotic ring covering the base of the cecum was discovered, and the appendix had perforated. There was a large collection of dishwater-like, foul-smelling fluid that extended deep into the space of Retzius (i.e. retropubic space). This collection was consistent with periappendiceal abscess. Its cultures grew out Escherichia coli, Pseudomonas aeruginosa, and Proteus mirabilis. The dome of the bladder was noted to be necrotic and ruptured, and in direct contact with the abscess. In summary, the general surgeon performed an appendectomy, drainage of the periappendiceal abscess, repair of the bladder perforation, resection of necrotic tissue of the abdominal wall, and abdominal washout prior to closure of the abdomen. Urology service was consulted intraoperatively for assistance in the management of bladder perforation via telephone.

Postoperatively, the patient remained intubated in critical condition, requiring low-dose norepinephrine infusion. Antibiotics were tailored to the cultures, with a regimen of clindamycin, cefepime, and metronidazole. On post-operative day 13, the patient’s urine output decreased significantly, and a repeat CT scan was performed, demonstrating free fluid in the abdomen likely representing urine, as well as persistent subcutaneous gas in the abdominal wall. The patient was taken back to the operating room, and it was found that she had progressive necrosis of the bladder wall with a perforation at the site of the initial repair and gross spillage of urine. There was also evidence of ongoing necrotizing fasciitis of the peritoneum and anterior abdominal wall, contributing to the continued septic shock. She underwent abdominal wall debridement, washout, and repeat repair of the bladder perforation. On post-operative day 19, the patient’s septic shock did not improve and care was withdrawn, and the patient died shortly thereafter.

## Discussion

Necrotizing fasciitis is a rare and predominantly polymicrobial, gas-forming infection that aggressively spreads along the fascial layer. It can rapidly lead to sepsis and death, if untreated [7]. Physical exam findings may include crepitus, diffuse tenderness, and rapidly spreading erythema. Early surgical debridement remains the key intervention for survival in addition to broad-spectrum antibiotics [8]. The empiric antibiotic regimen must cover anaerobic agents, and the initial recommended treatment is piperacillin-tazobactam plus vancomycin plus clindamycin [9]. Most frequently isolated bacteria from necrotizing fasciitis are Escherichia coli, Proteus mirabilis, Klebsiella pneumoniae, Staphylococcus aureus, and group D Streptococcus [10]. An intraoperative culture from our patient’s periappendiceal abscess revealed 2+ Proteus mirabilis, 2+ Escherichia coli, 1+ Pseudomonas aeruginosa.

Acute appendicitis has an incidence of 233/100,000 population and is most frequently seen in the 10-19 year old age group, and perforation is seen in approximately 25% of cases [11]. Although appendicitis is a common surgical emergency, associated necrotizing soft tissue infection is extremely rare and carries a high risk of mortality. It is predominantly associated with perforation of the appendix and delay in diagnosis [12]. One prospective study noted that 65% of patients with perforation of the appendix presented more than 48 hours after onset of symptoms [13]. Our patient presented four days after the onset of abdominal pain with symptom progression to feculent emesis. Acute perforated appendicitis poses a challenge for the clinician both in diagnosis and management. Clinical diagnosis of an acute abdomen may be evident by peritonitis on exam, but further imaging such as CT scan is essential in guiding specific management, especially when rare complications may be present. Our patient did not demonstrate crepitus or overlying skin changes on presentation. In addition, it is often difficult to distinguish other soft tissue infections from necrotizing fasciitis. A scoring guideline, Laboratory Risk Indicator for Necrotizing Fasciitis (LRINEC), has been suggested which is primarily based on laboratory values [14].

Complications from acute appendicitis vary widely, many of which are secondary to the effects of visceral perforation. They include bowel obstruction, abscess formation, uropathy, and gastrointestinal bleeding among others [15]. Our patient had a perforation of the appendix and subsequent periappendiceal abscess formation with gas-forming bacteria that caused necrotizing fasciitis of the abdominal wall. By the day of presentation, her bladder which was directly posterior to the abscess had also perforated. This was the main factor in her poor outcome, as spillage of urine noted on her repeat laparotomy lead to continued septic shock. Of note, the bladder perforation was not detected until the initial laparotomy was under way on the day of presentation. The initial CT scan did not reveal this finding, although it did demonstrate proximity of the abscess to the bladder as well as gas that encircled the bladder. Therefore, in a patient with perforated appendicitis with abscess formation, a genitourinary diagnostic modality such as a CT cystography may help with operative management.

Consideration of rare but serious complications of common diseases must be integrated into routine clinical decision-making. The emergency medicine physician assumes the challenging task of not only providing medical management for patients with a surgical abdomen but also consulting the appropriate subspecialties for an optimal outcome. This is especially difficult but equally important when definitive imaging modalities such as the CT scan are unable to reveal every relevant pathology affecting the patient.

## Conclusions

This was a rare case of necrotizing fasciitis and bladder perforation precipitated by acute perforated appendicitis with abscess formation. Even though the mortality rate of acute appendicitis has decreased with advances in medicine, late complications are much less studied and carry a high mortality rate. Our case presented such a patient with a poor outcome. If a patient’s physical exam demonstrates peritonitis, the bedside physician must have a high degree of suspicion for advanced complications of visceral perforation, including but not limited to abscess formation, necrotizing fasciitis, and perforation of periappendiceal structures such as the bladder.
